# A chemical accident cause text mining method based on improved accident triangle

**DOI:** 10.1186/s12889-023-17510-w

**Published:** 2024-01-02

**Authors:** Zheng Li, Min Yao, Zhenmin Luo, Xinping Wang, Tongshuang Liu, Qianrui Huang, Chang Su

**Affiliations:** 1 College of Safety Science and Engineering, Xi’an University of Science and Technology, Xi’an, 710054 China; 2 Institute of Management Science, Ningxia University, Yin’chuan, 750021 China; 3 College of Management, Xi’an University of Science and Technology, Xi’an, 710054 China

**Keywords:** Accident triangle, Accident classification, Risk factors, Text mining, K-means algorithms

## Abstract

**Background:**

With the rapid development of China’s chemical industry, although researchers have developed many methods in the field of chemical safety, the situation of chemical safety in China is still not optimistic. How to prevent accidents has always been the focus of scholars’ attention.

**Methods:**

Based on the characteristics of chemical enterprises and the Heinrich accident triangle, this paper developed the organizational-level accident triangle, which divides accidents into group-level, unit-level, and workshop-level accidents. Based on 484 accident records of a large chemical enterprise in China, the Spearman correlation coefficient was used to analyze the rationality of accident classification and the occurrence rules of accidents at different levels. In addition, this paper used TF-IDF and K-means algorithms to extract keywords and perform text clustering analysis for accidents at different levels based on accident classification. The risk factors of each accident cluster were further analyzed, and improvement measures were proposed for the sample enterprises.

**Results:**

The results show that reducing unit-level accidents can prevent group-level accidents. The accidents of the sample enterprises are mainly personal injury accidents, production accidents, environmental pollution accidents, and quality accidents. The leading causes of personal injury accidents are employees’ unsafe behaviors, such as poor safety awareness, non-standard operation, illegal operation, untimely communication, etc. The leading causes of production accidents, environmental pollution accidents, and quality accidents include the unsafe state of materials, such as equipment damage, pipeline leakage, short-circuiting, excessive fluctuation of process parameters, etc.

**Conclusion:**

Compared with the traditional accident classification method, the accident triangle proposed in this paper based on the organizational level dramatically reduces the differences between accidents, helps enterprises quickly identify risk factors, and prevents accidents. This method can effectively prevent accidents and provide helpful guidance for the safety management of chemical enterprises.

**Supplementary Information:**

The online version contains supplementary material available at 10.1186/s12889-023-17510-w.

## Background

China’s five most dangerous industries are coal mining, metal and non-metal mining, construction, and chemical and fireworks manufacturing [1, 2]. Especially in the chemical industry, major accidents occur more frequently. According to statistics, from 2016 to 2021, there were 1050 chemical accidents in China, resulting in 1330 deaths. These major accidents lead to severe casualties, economic losses, environmental pollution, and other consequences and substantially affect society’s harmonious and stable development. Scholars have been studying how to avoid accidents for a long time [3, 4].

Heinrich[5] collected many industrial accident records from insurance companies through statistical analysis of many casualties. He proposed the accident triangle: the ratio of death, serious injury, minor injury, and non-injurious accidents in enterprises is 1:29:300. The accident triangle represents there are similar risk factors behind serious accidents and minor accidents, which have been used to guide the safety management of enterprises for a long time [6, 7]. Many sectors, such as the railway sector in the UK [8], the industrial sector in Germany [9], and the mining sector in Australia [10], conduct analyses of near misses and accidents without significant consequences to reveal operator errors and system deficiencies. It is generally believed that when the number of minor accidents increases, the accident triangle predicts that the number of serious accidents also increases, which can encourage enterprises to enhance safety management to prevent major accidents effectively[11].

Based on Heinrich’s research findings, researchers from different engineering fields have also developed similar accident triangles [12]. The Bird accident triangle indicates that the ratio of serious or disabling injury, minor injuries, property damage accidents, and incidents with no visible injury or damage is 1:10:30:600 [13]. Tye-Pearson’s principle states that the ratio of fatal or serious injury, minor injuries, first-aid treatment injuries, property damage accidents, and narrowly avoided accidents is 1:3:50:80:400 [14]. The International Association of Oil and Gas Producers (OGP) has collected the safety accident data since 1985. About 50 members of the oil and gas organization participated in the annual benchmark testing process, continuously updating their accident triangles [15].

However, with the deepening of research, some scholars questioned the effectiveness of the Heinrich accident triangle [16]. It mainly includes the following two points: (1) the ratio of lower to higher severity accidents exists in the form of a “safety-triangle”; (2) similar causes underlie both high and low severity events [17].

Regarding the first criticism, Rebbit suggested that, given that Heinrich’s original data are not available, it is not possible to “verify or categorically refute” the specific ratio within the triangle. He further argued that the general way in which Heinrich categorized the safety accidents demarcated by severity (i.e., major, minor, and incident) makes it challenging to conduct replicable studies [18]. Marshall et al. stated that an analysis of occupational accidents across all the industries in Chile over 28-months shows that the ratio of fatal, serious and minor workplace accidents do not follow the ratio of accidents described in Heinrich accident triangle [19]. Yorio et al. studied the accident data from mines in the United States to confirm the predictive validity of the Heinrich accident triangle by checking if a certain number of accidents at a mine will produce a corresponding number of fatalities at the same mine. The results of their study did not match the figures described by Heinrich in his pyramid of accidents [17].

Regarding the second criticism, several studies have raised doubts about the assumption of similar causes, which determines whether enterprises focusing on minor accidents can effectively reduce the occurrence of major accidents [20–22]. Manuele argued that fatality and severe injury events often occur without any prior evidence or forewarning obtained through the analysis of less severe and near miss accidents [16]. Hale argued that the accident triangle has been abused and that preventing minor accidents will not automatically reduce serious accidents [23]. More and more evidence shows that companies with a very low incidence of minor accidents will also encounter serious accidents [24]. In the United States, although the incidence of non-fatal accidents in the entire workforce has decreased by 51% over the past decade, fatal accidents have only decreased by 25.5% [25]. Although studies have supported the notion that safety accidents delineated by degree have distinct causes [26] others found consistent causes between low and high severity events [8].

The traditional accident triangle usually analyzes personal injury accidents [27], dividing the accidents into death, serious injury, minor injury, and non-injury accidents for risk factor analysis. It rarely analyses property loss and environmental protection accidents, failing to identify some hidden risk factors [28]. The accident classification is based mainly on the consequences and types of accidents. The accident gradation is based on the accident severity. Its purpose is to provide a basis for preparing accident investigation reports, handling the person responsible for the accident, legal compensation, and other matters [29]. According to the “Byelaw Governing Reporting, Investigation and Handling of Production Safety Accidents” in China, accidents are divided into particularly serious accident, major accident, serious accident, and accident of minor seriousness in terms of bodily injuries and deaths or direct economic losses resulted in by the production safety accidents [30]. Based on the accident triangle, Sinopec proposes a three-level accident classification model from the perspective of organizational structure: group company-level accidents, recorded accidents, and accidents that require upgraded management. The accident gradation is more in-depth and detailed, and the requirements for casualties and direct economic losses are more strict [29].

The accident triangle is an adequate rule-of-thumb for safety planning; like any theory, it must be tested and updated. Industries need a set taxonomy for hazards/accidents and data to calibrate it [31]. Chemical enterprises are characterized by large scale, multiple organizational departments and levels, harsh production process conditions, complex equipment, and numerous risk factors [32, 33]. It is necessary to conduct a comprehensive risk factor analysis for chemical enterprises. Given the highly influential and debate of the accident triangle, additional research on the topic is important. This paper will test and update the accident triangle by developing a new accident classification method.

Although the industries differ, the accidents have similar trajectories [34]. Learning from the accident is considered the critical link to preventing future injuries [35], focusing on determining the root cause of the accident [36]. Currently, this work mainly depends on the judgment of domain experts, which is subjective and time-consuming. Enterprises have accumulated many safety accident reports. These unstructured text forms increase the difficulty of tacit mining knowledge. In recent years, data analysis in accident reports has provided a new way to research the causes of accidents [37]. As a branch of data analysis, text mining can extract unknown but valuable information and knowledge from unstructured text sets, involving knowledge in multiple fields such as artificial intelligence, machine learning, and natural language processing (NLP) [38]. It is currently a research hotspot in text information processing [39]. Since Feldman et al. [40] first proposed the concept of text mining in 1995, the development of text mining technology has become mature. It has been widely applied in fields such as biomedical [41], consumer behavior [42], emotional analysis [43], coal mining production [44, 45], transportation [46], and construction [47].

In the field of engineering safety management, the dispersion, diversity, and massive nature of safety data have led to difficulties in collecting and processing safety texts, thereby promoting the application of text mining technology [48]. In recent years, a few scholars have utilized text mining technology to extract key accident features and risk factors from accident investigation reports, fully leveraging the role of accident investigation reports in summarizing experiences and lessons learned and curbing accident risks [49, 50]. For example, Gao et al. [51] developed a verb-based text mining method that extracted the causes and results of 945 car traffic accident reports, which helps to understand the true causes of traffic accidents. Qiu et al. [52] combined text mining technology with complex networks to explore the causal mechanisms of coal mine accidents. Through text mining of 307 accident reports, 52 main accident causal factors were identified, and a coal mine accident causal network was constructed based on strong association rules between factors, providing a new perspective for identifying accident causes and their complex interaction mechanisms from accident report data. Esmaeili et al. [53] used text mining technology to obtain accident attribute feature values from over 1000 construction accident reports and conducted statistical analysis, ultimately identifying the risk factors of construction accidents. Raviv et al. [54] analyzed 212 near-miss and accident reports on tower cranes using text mining and k-means algorithms and found that technical failures are the most dangerous risk factor in tower cranes.

In the field of chemical safety management, work on anomaly detection [55], ontology-based knowledge acquisition [56], and process alarm prediction [57] have been undertaken based on accident texts. Despite such work, no existing method meets the demands of both universality and accuracy, and there still needs to be an efficient, convenient universal tool for extracting risk factors from chemical accident cases.

To solve the problem that serious accidents are difficult to eradicate, we select chemical enterprises with many risk factors for empirical research to explore the factors causing severe accidents. Firstly, based on the accident triangle, combined with the characteristics of large-scale, multiple organizational departments and levels of the chemical industry, the organizational-level accident triangle has been proposed. Try to determine the distribution law of risk factors in chemical enterprises through a new classification method. Secondly, this paper uses text mining technology to conduct text clustering analysis on unstructured accident reports, quickly find the risk factors behind different accidents, and solve the disadvantages of time-consuming and labor-consuming finding accident causes in the past.

## Methods

**Fig. 1:**
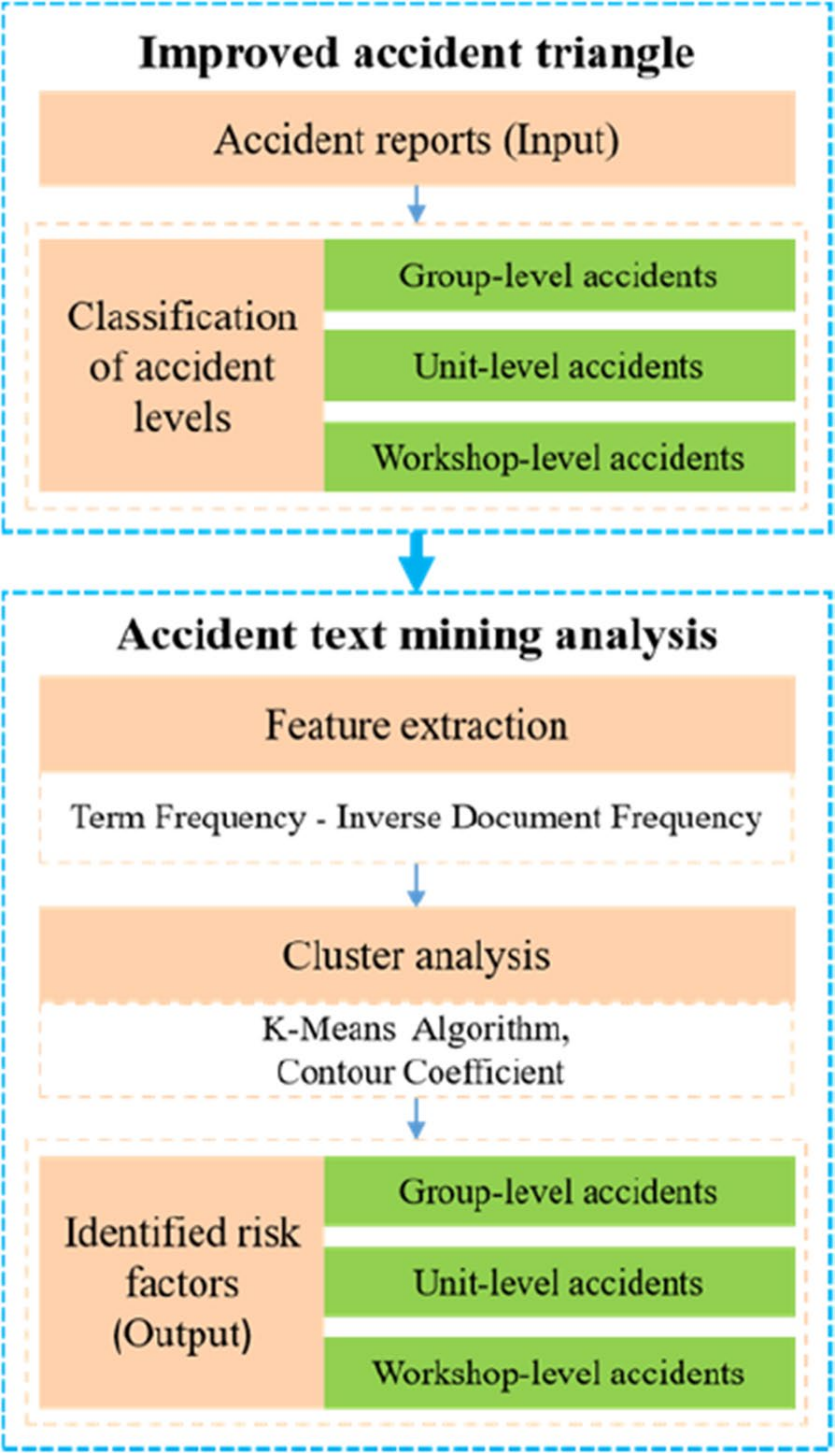
Overall Flowchart of the proposed model

This study comprehensively considers personal injury accidents, property loss accidents, and environmental accidents in enterprises. It proposes an accident triangle based on the organizational level, which classifies accidents into group-level accidents, unit-level accidents, and workshop-level accidents. The collected accident reports are model input, and each accident is classified into levels using the organizational level accident triangle. Then, text mining technology is used to cluster and analyze the causes of accidents at different organizational levels by identifying the various risk factors contained in accidents at different organizational levels, namely the model output, to help enterprises formulate targeted risk control measures. The overall flow of this model is shown in Fig. 1.

### Improved accident triangle

The traditional accident triangle usually analyzes personal injury accidents and classifies the accident severity into four categories: death, serious injury, minor injury, and non-injury. However, enterprises will also have accidents of different natures in daily production, such as production, quality, environmental, and property loss accidents [58]. This study will propose a more comprehensive accident classification method that divides accidents into personal injury accidents, property loss accidents, and environmental protection accidents, addressing the drawbacks of traditional accident triangles that cannot analyze all risk factors. For example, traditional accident triangles focus on analyzing personal injury accidents, often categorizing property loss accidents and environmental accidents roughly as non-injury accidents or near misses, which is not conducive to further accurately identifying the risk factors that cause accidents.

**Fig. 2:**
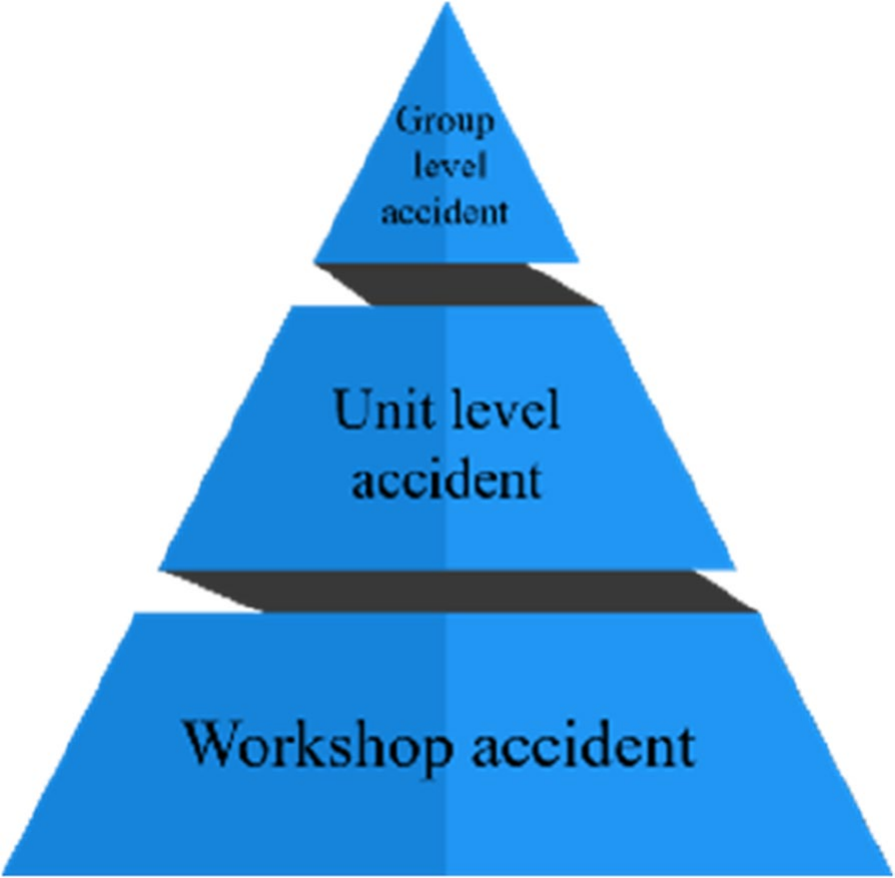
The organization-level accident triangle

This study focuses on identifying accident risk factors in China’s chemical industry, improving the accident triangle, and reclassifying accidents from the organizational level. The organization-level accident triangle divides enterprise accidents into group-level, unit-level, and workshop-level. In risk factor mining for different accident levels, analyze the ratio and causes of different accident levels. Accidents at all levels are classified according to severity; the definitions and classification standards for group-level, unit-level, and workshop-level accidents are shown in Additional file 1. The bottom of the organization-level accident triangle is the number of workshop-level accidents; the middle refers to unit-level accidents; the top is the number of group-level accidents (Fig. 2).

**Fig. 3:**
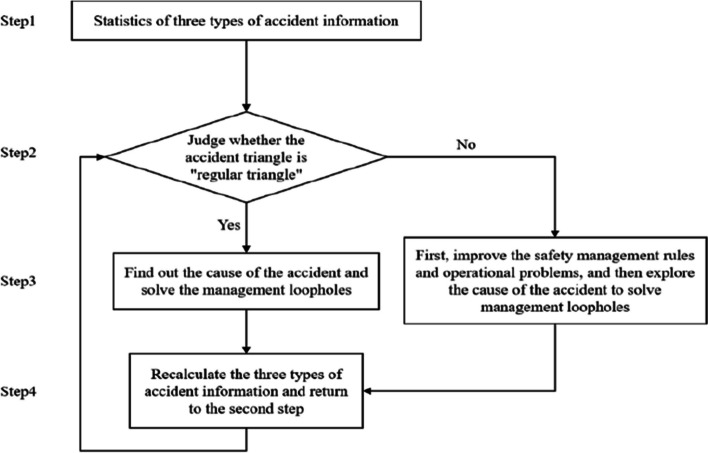
Analysis flowchart for the use of the organizational-level accident triangle

Applying the organizational-level accident triangle includes the following processes: In Step 1, the relevant information of three levels of accidents is counted, and the accident management account is updated in time. Step 2, whether the accident triangle based on the organization level is in the state of a “positive triangle” or “inverted triangle” is checked by counting the frequency of occurrence of three levels of accidents. Accidents at different levels are divided according to severity. Based on practical experience, there are far more minor accidents than serious ones. Then, the number of workshop-level accidents should be the largest, the number of unit-level accidents should rank second, and the number of group-level accidents should be the least. If the proportion of accidents at different levels meets this requirement, it is considered in the positive triangle state. Otherwise, it is in the inverted triangle state. Step 3, if in the “positive triangle” state, indicates that the design of enterprise safety rules and regulations is reasonable. Further, it analyzes various accidents, determines safety management loopholes, and eliminates relevant risk factors. Otherwise, it indicates that the design and implementation of enterprise safety management rules and regulations are unreasonable. The enterprise needs to solve this problem and use text mining technology to ascertain the causes of accidents, reveal management loopholes, and eliminate risk factors. Step 4 is accident statistics for the enterprise again, followed by the second step to start a new round of analysis. The process is shown in Fig. 3.

This paper uses the Pearson correlation coefficient method to analyze the correlation among organizational-level, unit-level, and workshop-level accidents. The correlation coefficient is expressed in R, with a value range of 0 to 1.

1 $$R=\frac{n\sum XY - \left(\sum X\right)\left(\sum Y\right)}{\sqrt{\left[n\sum {X}^{2} -{\left(\sum X\right)}^{2}\right]\left[n\sum {Y}^{2 }-{\left(\sum Y\right)}^{2}\right]}}$$

Where, *n* refers to the total number of accidents, while *X* and *Y* refer to the number and average value of accidents at different levels. The closer the absolute value of *R* is to 1, the better the correlation.

### Text mining analysis of accidents at different organizational levels

Text mining technology has been widely used to study accident causes. Through extensive research of accident reports, text mining can better understand the causes of accidents and significantly improve the accuracy of accident prediction [59].

**Fig. 4:**
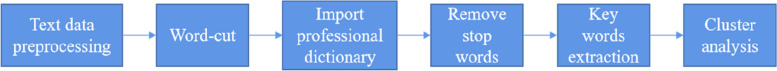
Text mining analysis process

In recent years, the analysis of incident/accident data using data mining techniques and algorithms has attracted much attention among researchers [60, 61], which promotes the birth and development of data-mining technology [62]. Many data mining technologies, such as Support Vector Machine [63], classification analysis [64], cluster analysis [65], association analysis [66], chi-square automatic interaction detection [67], and Bayesian networks [68], are used to identify the hidden patterns and structures in the safety database. This article uses the text clustering method to identify risk factors in accident text, and the analysis process is shown in Fig. 4.

#### Extract keywords

Text keyword extraction requires pre-processing technology to convert the text into a form the computer can recognize. This paper uses the Jieba Chinese word segmentation tool to segment and label accident records. To identify professional terms and idioms and ensure that these words are not segmented, compiled a professional dictionary according to the vocabulary of the coal chemical industry and the expression characteristics of enterprise safety officers on potential safety hazards and accident records. Stop words are words and tonal symbols that frequently appear in the text but have no functional meaning and do not help analyze the main idea of the text. These meaningless words can be deleted by importing the stop word list. After getting keywords and assigning weight to each keyword, the Term Frequency Inverse Document Frequency (TF-IDF) is usually used as a feature evaluation function for feature extraction [69]. TF-IDF is expressed as:

2 $$TF-{IDF}_{i,j}= {tf}_{i,j}\times {idf}_{i}$$

3 $${tf}_{i,j}=\frac{{n}_{i,j}}{{\sum }_{K}{n}_{k,j}}$$

4 $${idf}_{i}=\mathrm{log }\frac{|D|}{1+|\left\{j:{t}_{i}\in {d}_{j}\right\}|}$$

Where, ${n}_{i,j}$ denotes the number of occurrences of the keyword ${t}_{i}$ that appears in the accident record document ${d}_{j}$, and $\sum_{K}{n}_{k,j}$ is the number of all keywords in the accident record document ${d}_{j}$ $|D$| represents the total number of accident record documents, and $\left|\left\{j:{t}_{i}\in {d}_{j}\right\}\right|$ is the number of documents containing keyword ${t}_{i}$, to avoid this item being zero and the divisor being zero, it is generally expressed as $1+\left|\left\{j:{t}_{i}\in {d}_{j}\right\}\right|$.

TF denotes the number or frequency of a word in the article. The word is essential if a keyword appears multiple times in an accident record document. IDF represents the recognition degree of a keyword in the accident record document. The larger IDF value means the keyword is essential in this document and vice versa. TF-IDF integrates the advantages of TF and IDF [70].

#### Text clustering analysis

Clustering is widely applied in machine learning and data mining as a standard data research method. The common clustering methods are the minimum distance within the group and the maximum distance between groups [71]. The basic idea of the *K*-means algorithm proposed by MacQueen [72] is to divide all objects into *K* clustering centers according to the nearest principle. The similarity between texts is measured by Euclidean distance. Before *K*-means clustering, we need to determine the K value and the number of clusters. Randomly obtain *K* initial cluster centers and iterate the average similarity between documents until the optimal solution is derived.

*K*-means clustering algorithm takes the sum of squares errors (SSEs) as the objective function to minimize the SSEs between texts in *K* clusters. The cluster center ${e}_{i}$ of cluster ${E}_{i}$ can be expressed as:

5 $${e}_{i}=\frac{1}{{n}_{i}}\sum_{n\in {E}_{I}}x$$

The SSE between texts is calculated as follows:

6 $$SSE = \sum_{i =1}^{K}\sum_{n\in {E}_{i}}cos{\left({e}_{i},x\right)}^{2}$$

Where, $x$ represents the text object, ${E}_{i}$ is the *i*^th^ cluster, ${n}_{i}$ denotes the number of samples therein, and ${e}_{i}$ is the center of cluster ${E}_{i}$.

Peter [73] first proposed the contour coefficient to calculate the *K* value in *K*-means text clustering, which can judge the text clustering effect. Averaging the contour coefficients of all vectors is the contour coefficient of the cluster. The contour coefficient’s absolute value is not greater than 1. The larger the average value of the contour coefficient, the better the text clustering effect.

## Results

### Database

The data in this paper comes from a large coal chemical enterprise in China. The group has more than a dozen secondary units, such as a coking plant, methanol plant, olefin plant, and power company. The company has formulated safety management regulations by relevant national laws, such as “Classification Standard for Casualty Accidents of Enterprise Employees” (GB6441), “Identification of Labor Ability and Disability Grade of Employees Caused by Industrial Injury and Occupational Disease” (GB/T16180), “Byelaw Governing Reporting, Investigation and Handling of Production Safety Accidents” (An order by PRC State Council No. 493), and conducted daily safety management and accident investigation under regulations.

**Fig. 5:**
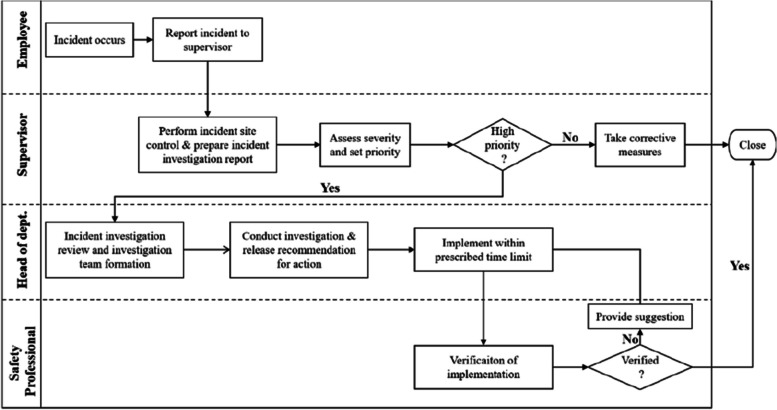
Proposed workflow through the accident investigation module

The workflow for accident investigation is shown in Fig. 5. During the accident investigation, the safety technician and safety supervisor are the backbone of promoting the smooth progress of the accident investigation. To ensure the objectivity and authenticity of the accident report, the responsible person related to the accident cannot be a member of the accident investigation team. After the accident investigation, the safety department will prepare the accident report as required according to the collected data.

The database used in this paper consists of 484 accident records generated by SMS from 2015 to 2020; Additional file 2 lists information from two of the accident reports. The accident records contain all accident information of the enterprise, including accident position, accident time, accident level, accident nature, consequence degree, and accident process.

The database used in the work presented here records the accident information of the sample enterprises in a certain period of actual production. Because all secondary units conducted daily safety management under the safety management rules and regulations formulated by the group safety department, all safety managers and on-site operators unified the rules and regulations. The accident data can reflect various risk factors in enterprises, and it is feasible to use the method proposed in this paper to identify enterprise risk factors.

### Correlation analysis of accidents at different organizational levels

A statistical analysis of the company's accidents from 2015 to 2020 is conducted from the organizational level perspective (Table 1). The ratio of group-level, unit-level, and workshop-level accidents is 1:3:12; this division dramatically reduces the difference between accidents and helps find common causes at different organizational levels.

**Table 1:** Annual accident statistics of sample enterprises at different organizational levels

Year	Group-level	Unit-level	Workshop-level
2015	5	23	60
2016	4	11	79
2017	4	11	70
2018	5	11	67
2019	6	12	51
2020	7	12	46

**Fig. 6:**
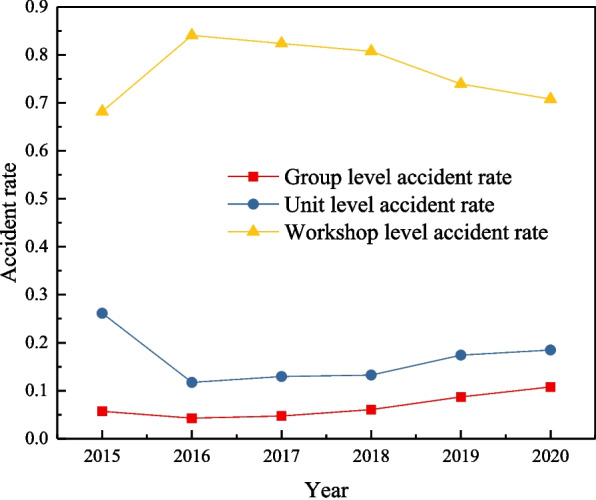
Trends of accident rate at different organizational levels

To more intuitively display the annual change trend of accidents at different organizational levels, a trend chart of accident rate change has been prepared, as shown in Fig. 6. The trend of change in unit-level and group-level accident rates is highly similar. In contrast, the changing trend in both is opposite to that in the workshop-level accident rate. Spearman correlation analysis is performed among the group, unit, and workshop accident rates, and the results are shown in Table 2. The correlation coefficient between the group-level accident rate and the unit-level accident rate is 0.657, which passes the significance test at 0.05,, showing a significant positive correlation between the unit-level accident rate and group-level accident rate. In enterprise safety management, rectification measures should be implemented for unit-level accidents. Reducing the unit accident rate can also reduce group-level accidents and overcome the difficulty of finding causal factors because the number of group-level accidents is too small. The group-level and unit-level accident rates negatively correlate with the workshop-level accident rates; the correlation coefficients are -0.594 and -0.857, respectively, and pass the significance test at the level of 0.05. The enterprise accident data are divided into different levels of accidents; when the proportion of group-level and unit-level accidents is relatively small, the proportion of workshop-level accidents is relatively large. On the other hand, it can also be explained that when workshop-level accidents occur frequently, staff and managers will spend more time paying attention to safety problems and constantly solve the neglected risks arising from minor accidents to avoid more severe accidents.

**Table 2:** Correlation analysis of accident rates at different organizational levels

		Group-level accident rate	Unit-level accident rate	Workshop accident rate
Group-level accident rate	Correlation coefficient	1		
	Significance	0		
Unit-level accident rate	Correlation coefficient	0.657	1	
	Significance	0.038	0	
Workshop accident rate	Correlation coefficient	-0.594	-0.857	1
	Significance	0.016	0.008	0

### Text mining analysis

The work divides enterprise accidents into group, unit, and workshop levels based on the organizational-level accident triangle. The characteristics and risk factors of the three levels of accidents will be found by text mining. This paper will conduct text mining on 484 accident records, including 373 accident records at the workshop level, 80 at the unit level, and 31 at the group level.

#### Extract keywords

The TF-IDF method assigns weight to accident keywords at all levels. The TF-IDF values of each keyword are arranged in descending order, as illustrated in Table 3, 4 and 5. The top 30 accident keywords at all levels are displayed. The keywords with a high weight of workshop-level accidents include gasifier, boiler, inspection, induced draft fan, compressor, economizer, liquid level, and tripping. It indicates that workshop-level accidents are mostly related to this equipment. The accident records demonstrate that the keywords with high weight ranking can accurately reflect the frequent workshop-level accidents.

**Table 3:** Weighted values of keywords in workshop-level accident keywords (top 30)

No	Keywords	TF-IDF	No	Keywords	TF-IDF	No	Keywords	TF-IDF
1	Gasifier	0.1041	11	Flow	0.0472	21	Furnace shutdown	0.0308
2	Boiler	0.0715	12	Overhaul	0.0458	22	Surge	0.0307
3	Central Control Room	0.0698	13	Discover	0.0423	23	Gasification	0.0302
4	Load	0.0601	14	Interlock	0.0404	24	Scheduling	0.0293
5	Check	0.0590	15	Pressure	0.0386	25	Pulverized coal	0.0287
6	Induced draft fan	0.0589	16	Valve	0.0331	26	Meter	0.0283
7	Fan	0.0550	17	Start-up	0.0322	27	Compressor	0.0280
8	Running	0.0531	18	Trip	0.0322	28	Conversion	0.0280
9	Workshop	0.0518	19	Switch	0.0319	29	Shut down	0.0277
10	Liquid level	0.0518	20	Stop	0.0319	30	Economizer	0.0257

**Table 4:** Weighted values of keywords in unit-level accidents (top 30)

No	Keywords	TF-IDF	No	Keywords	TF-IDF	No	Keywords	TF-IDF
1	Scene	0.1000	11	Pipeline	0.0437	21	Display	0.0306
2	Central control room	0.0954	12	Compressor	0.0427	22	Stop	0.0304
3	Coke oven gas	0.0795	13	Load	0.0393	23	Pressure relief	0.0304
4	Interlock	0.0729	14	Liquid level	0.0390	24	Supercharger	0.0304
5	Gasifier	0.0604	15	Slag lock bucket	0.0376	25	Synthesis	0.0300
6	Scheduling	0.0544	16	Running	0.0372	26	Close	0.0300
7	Workshop	0.0497	17	Work	0.0371	27	Methanol	0.0296
8	Notice	0.0490	18	Pressure	0.0365	28	Main operation	0.0289
9	Induced draft fan	0.0447	19	Overhaul	0.0320	29	Water seal	0.0286
10	Reactor	0.0441	20	Flow	0.0313	30	Check	0.0285

**Table 5:** Weighted values of keywords in group-level accidents (top 30)

No	Keywords	TF-IDF	No	Keywords	TF-IDF	No	Keywords	TF-IDF
1	Reactor	0.1028	11	Workshop	0.0470	21	Fan	0.0357
2	Scene	0.0777	12	Be on duty	0.0454	22	Conversion	0.0356
3	Coke quenching car	0.0648	13	Central control room	0.0449	23	Boiler	0.0356
4	Pressure	0.0640	14	Rotating speed	0.0426	24	Electric machinery	0.0355
5	Liquid level	0.0623	15	High-pressure cylinder	0.0424	25	Coke quenching	0.0349
6	Main operation	0.0598	16	Stop	0.0399	26	Lean liquid pump	0.0349
7	Scheduling	0.0548	17	Pure benzene	0.0399	27	Surge	0.0330
8	Coking plant	0.0542	18	Feedwater pump	0.0377	28	Propylene	0.0321
9	Compressor	0.0532	19	Check	0.0365	29	Lubricating oil	0.0318
10	Vibration	0.0504	20	Loading	0.0362	30	Pipeline	0.0317

The keywords with a high weight of unit-level accidents include coke oven gas, interlock, gasifier, induced draft fan, reactor, and pipeline, suggesting that unit-level accidents are mostly related to the above keywords. The accident records show that the causes can be classified as coke oven gas leakage, pipeline rupture, induced draft fan component damage, and excessive fluctuation of process parameters.

The keywords with high weights among group-level accident reports include reactor, coke quenching vehicle, pressure, liquid level, coking plant, compressor, vibration, and pure benzene, implying that group-level accidents are mostly related to the above keywords. By comparing the accident records, group-level accidents are found to be mainly death and severe injury accidents caused by toxic substance leakage, mechanical injury and safety barrier damage, environmental accidents caused by ammonia and raw gas leakage, shutdown accidents arising from equipment damage, and excessive fluctuation of process parameters.

### Text clustering analysis

**Fig. 7:**
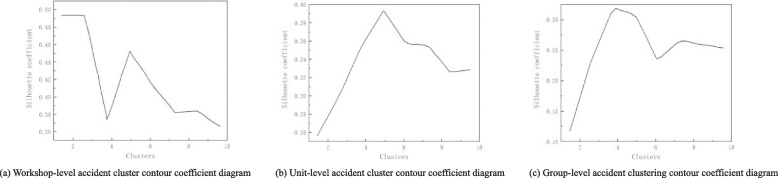
Clustering contour coefficients of accidents at different levels

The contour coefficient is calculated for the text data of accident records at different levels (Fig. 7). The corresponding contour coefficient value is the largest when the K values of workshop-level, unit-level, and group-level accident clusters are 5, 5, and 4. The optimal number of clusters for K-means text clustering of workshop-level, unit-level, and group-level accidents are 5, 5, and 4, respectively.

**Fig. 8:**
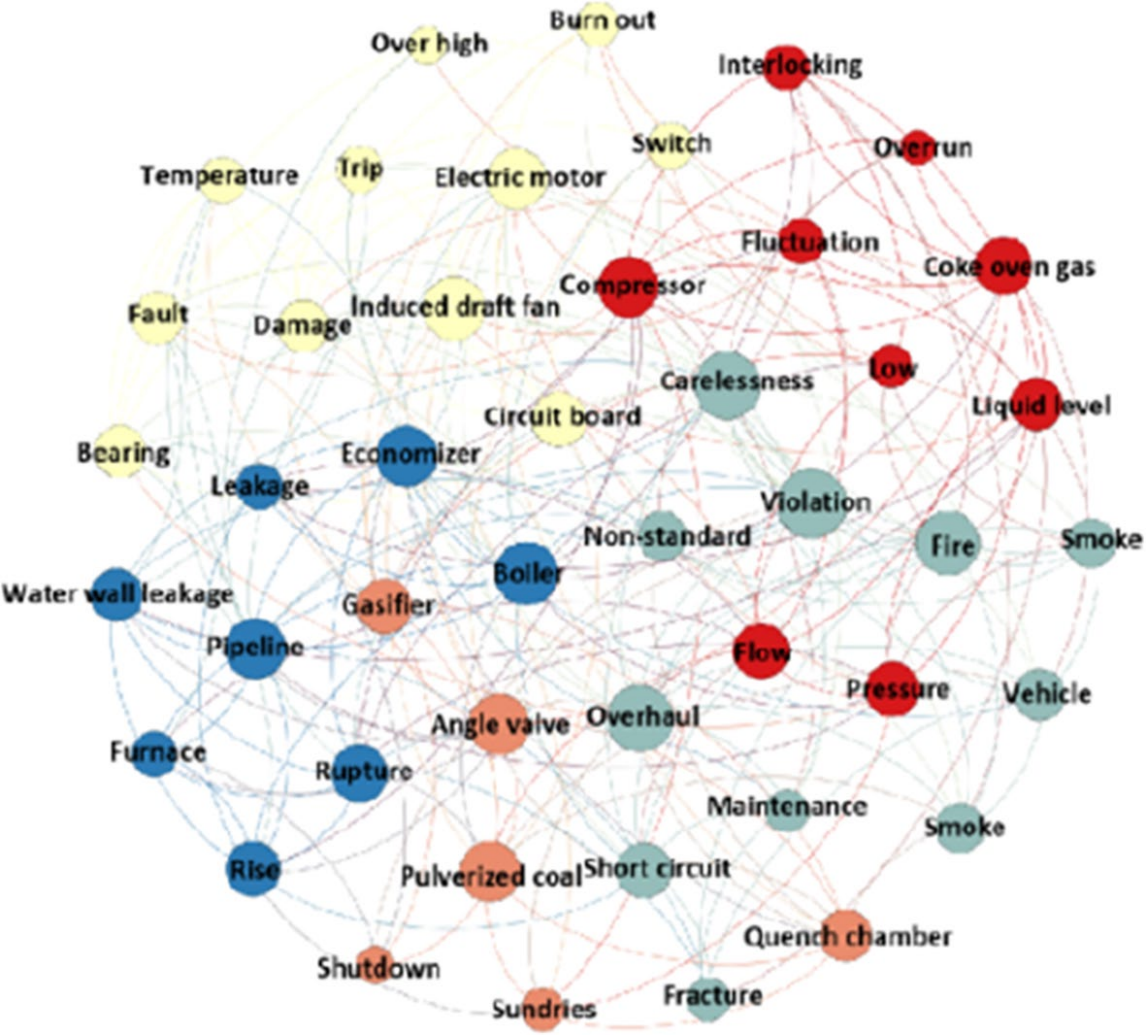
Visual clustering result of workshop-level accidents

Programming Python conducts K-means cluster analysis on accident records at different levels. The clustering result of workshop-level accidents is shown in Fig. 8; see Additional file 3 for detailed results.

The accident records in the five clusters are 181, 84, 41, 40, and 27, respectively. The accident type, high-frequency keywords, and accident cause analyses of each cluster are displayed in Table 6. The accident types of cluster 0 are mainly personal injury accidents; the main reason is unsafe personal behavior, and the unsafe state of things causes only a few accidents. Cluster 1 is mainly the production accident of shutdown and maintenance caused by the damage of induced draft fan, motor, and other equipment components. Cluster 2 primarily refers to shutdown and maintenance accidents caused by economizer leakage and pipeline rupture. Cluster 3 mainly represents shutdown accidents caused by excessive fluctuation of process parameter values and environmental accidents caused by hazardous substance leakage. Cluster 4 mainly refers to equipment-tripping accidents caused by untimely cleaning of sundries or malfunction, which also reflects the management vulnerability of the enterprise in terms of cleanliness and hygiene.

**Table 6:** Cluster analysis of workshop-level accidents

Cluster no	Accident types	High-frequency keyword	Main causes of accidents
0	Traffic accident, fire accident, mechanical injury accident, fall down accident, scald accident	Carelessness, vehicle, collision, fracture, fire, smoke, short circuit, crush injury, overhaul, violation, non-standard, maintenance, disassembly, cleaning, safety rope, slip, fall, high temperature, scald	Weak safety awareness, failure to take protective measures, non-standard operation, untimely communication, untimely equipment maintenance, circuit short circuit, and pipeline leakage
1	Production accident	Induced draft fan, electric motor, circuit board, bearing, switch, damage, fault, temperature, over high, burn out, trip, stop	Equipment failure, induced damage to draft fan parts, electric motor damage, over-temperature, switch trip
2	Production accident	Low temperature, high temperature, Economizer, boiler, pipeline, water wall leakage, rupture, leakage point, water level, rise, furnace, pressure, rise, boiler shutdown, maintenance	Economizer leak, pipe rupture, water wall rupture
3	Production accident, environmental pollution accident	Compressor, coke oven gas, flow, pressure, liquid level, too low, rise, fluctuation, interlocking, overrun, trip, shutdown, flare, black smoke, alarm	Significant fluctuation of gas flow/ liquid level/ pressure/ temperature, excessive vibration amplitude, damage to compressor components, pipe blockage, and flange leakage
4	Production accident	Angle valve, pulverized coal, gasifier, quench chamber, sundries, trip, shutdown	Untimely removal of sundries, malfunction, and excessive fluctuation of process parameters

In the workshop-level accidents cluster, the proportions of personal injury, property loss, and environmental accidents are 29%, 69%, and 2%. The leading causes of personal injury accidents are weak safety awareness of employees, failure to take protective measures, illegal operation, or nonstandard operation. The leading causes of property loss accidents are fire caused by a short circuit, untimely cleaning of sundries, and interlocking shutdown caused by equipment failure, leakage, and excessive fluctuation of process parameters. The leading cause of environmental accidents is the excessive discharge of pollutants caused by equipment failure and leakage.

**Fig. 9:**
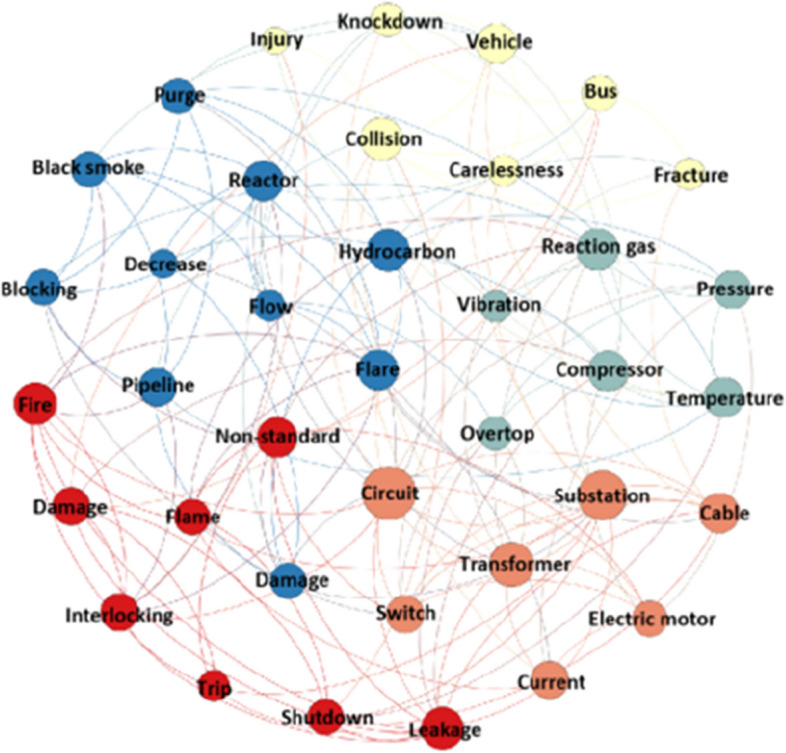
Visual clustering result of unit-level accidents

The cluster result analysis of unit-level accidents is shown in Fig. 9, and accident cause analyses of each cluster are displayed in Table 7.

**Table 7:** Cluster analysis of unit-level accidents

Cluster No	The accident types	High-frequency keyword	Main causes of accidents
0	Production accident, environmental pollution accident	Hydrocarbon, flare, black smoke, reactor, pipeline, purge, blocking, flow, liquid level, decrease, locking	Pipeline blockage, device damage
1	Fire accident, production accident, mechanical injury accident, scald accident	Non-standard, leakage, fire, smoke, flame, interlocking, shutdown, damage, trip	The operations are not standardized, and protective measures are not taken
2	Production accident	Circuit, substation, transformer, cable, switch, trip, current, high, electric motor	Short circuit, equipment power loss, voltage fluctuation, power trip
3	Traffic accident	Collision, vehicle, carelessness, bus, knockdown, fracture, injury	Lack of safety awareness and failure to comply with traffic rules
4	Production accident	Reaction gas, compressor, temperature, pressure, vibration, differential pressure, liquid level, overtop, too fast, interlock, trip	Excessive temperature / pressure / liquid level / vibration fluctuation

Cluster 0 mainly includes shutdown maintenance and environmental accidents caused by pipeline blockage and device damage. Cluster 1 mainly denotes personal injury accidents caused by employees’ non-standard operation or failure to take protective measures. Cluster 2 mainly refers to shutdown accidents caused by power system faults such as short-circuiting and substation tripping. Cluster 3 mainly denotes traffic accidents. Cluster 4 mainly refers to equipment tripping accidents caused by excessive fluctuation of process parameters.

Among unit-level accidents, personal injury, property loss, and environmental accidents accounted for 23%, 61%, and 16%, respectively. The leading cause of personal injury accidents is the non-standard operation of employees. The leading causes of property loss accidents are equipment failure, excessive fluctuation of process parameters, pipeline blockage, and leakage. The leading causes of environmental accidents are the excessive emission of harmful gases caused by substation failure, line short circuits, and control system failure.

**Fig. 10:**
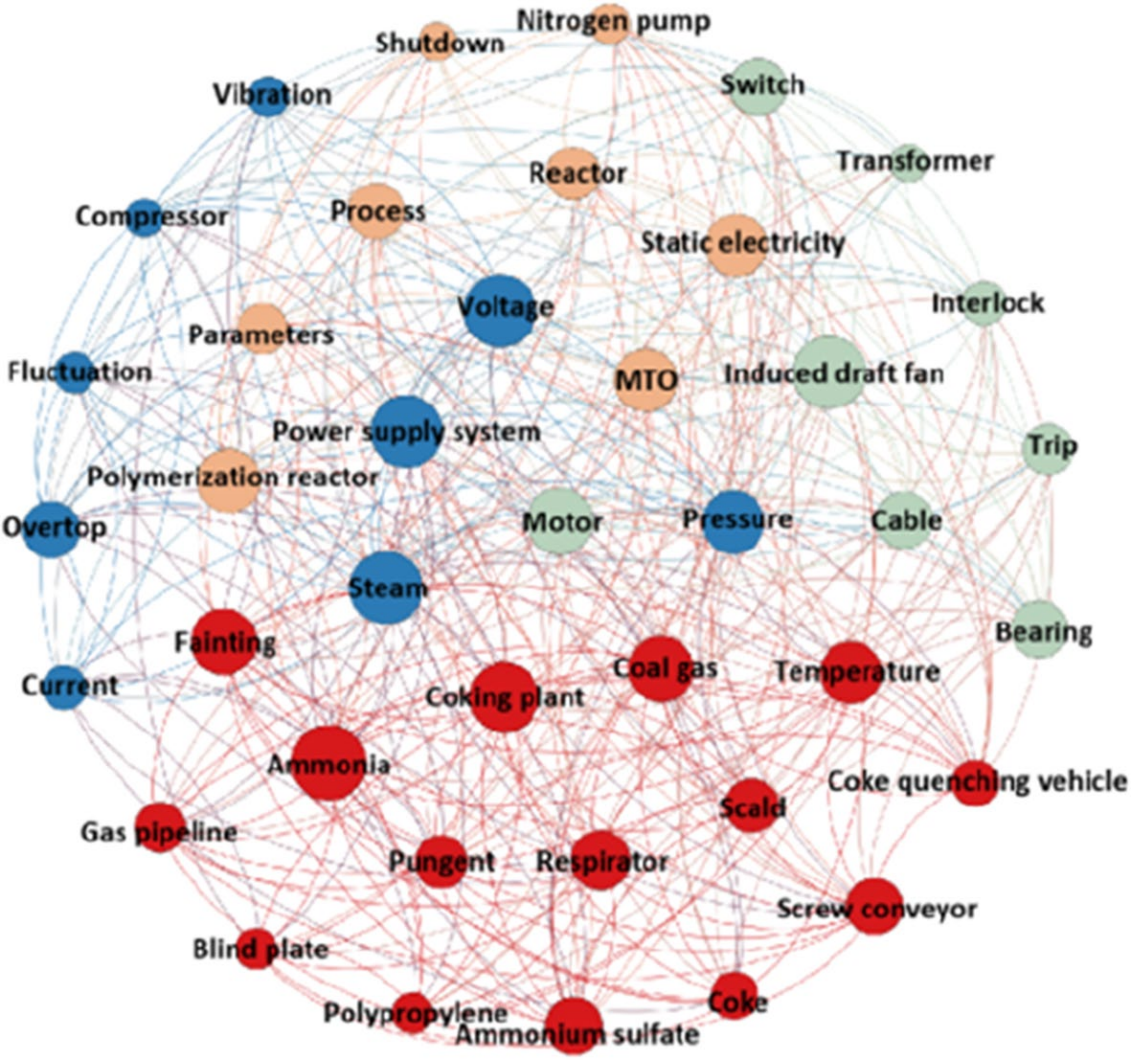
Visual clustering result of group-level accidents

The cluster result analysis of group-level accidents is shown in Fig. 10, and accident cause analyses of each cluster are displayed in Table 8.

**Table 8:** Cluster analysis of group-level accidents

Cluster No	The accident types	High-frequency keyword	Main causes of accidents
0	Poisoning accident, explosion accident, mechanical injury accident, scald accident, environmental pollution accident, product quality accident	Coking plant, coal gas, ammonia, temperature, respirator, fainting, ammonium sulfate, coke quenching vehicle, scald, pungent, coke, screw conveyor, gas pipeline, blind plate, polypropylene, death	Weak safety awareness, failure to take protective measures, illegal operation, imperfect product quality management system
1	Production accident	Power supply system, voltage, steam, pressure, temperature, overtop, vibration, current, fluctuation, compressor, blockage	Excessive temperature/ pressure/current/vibration fluctuation and pipe network blockage
2	Production accident	Induced draft fan, motor, cable, bearing, temperature, trip, interlock, transformer	Equipment components damaged, short circuit, switch trip
3	Production accident	Polymerization reactor, static electricity, process parameters, MTO reactor, pressure, temperature, vibration, nitrogen pump, shutdown	Equipment components damaged, static electricity, excessive pressure/ temperature/vibration fluctuation, and accumulated materials are not cleaned timeously

Cluster 0 mainly refers to personal injury accidents caused by unsafe factors such as employees’ weak safety awareness and illegal operations. Cluster 1 mainly denotes equipment shutdown accidents caused by excessive fluctuation of process parameters. Cluster 2 mainly indicates equipment tripping accidents caused by damage and short-circuiting. Cluster 3 mainly involves production accidents, but many causes include damage to equipment components, excessive fluctuation of process parameters, and untimely removal of sundries. The clustering effect is poor due to the small number of accidents.

In the group-level accidents cluster, personal injury, property loss, and environmental accidents accounted for 22%, 64%, and 14%, respectively. Personal injury accidents cause death and severe injury, and the main risk factors are illegal operations and failure to take protective measures. The main risk factor of property loss accidents is the failure of the compressor, separator, fan, and transformer. The main risk factor of environmental accidents is the leakage of pipes, flanges, and valves.

The clustering results show that the most significant proportion of accidents at the three levels is human factors, such as insufficient safety awareness of employees, illegal operations, and failure to inspect and repair equipment as required. It indicates that enterprises urgently need to train employees in safety knowledge and skills in future safety management. The leading causes of workshop-level accidents are equipment damage and pipeline leakage. It is necessary to conduct targeted maintenance and replacement of equipment components, such as induced draft fans, compressors, and economizers, and establish an assessment mechanism for regular maintenance. Power system faults account for a high proportion of unit-level and group-level accidents. Such accidents occur infrequently, but the degree of harm is enormous; it is necessary to strengthen the operation and maintenance of such power systems.

### Practical application

#### Improvement measures

Based on the risk factors identified by cluster analysis results, the enterprise has rectified activities from the perspective of unsafe human behavior and the unsafe state of objects. Due to inadequate safety awareness, irregular operations, and failure to take proper protective measures among employees, the vast majority of accidents have occurred. The company has established a safety inspection team directly responsible for senior leaders, providing two months of safety knowledge and technical training for all employees. The safety inspection team evaluates the training results of employees, and employees who fail the assessment are not allowed to take up their positions. In addition, an online knowledge learning and testing system has been designed based on different departments and job responsibilities. Employees can log in to the system through their mobile phones for problem-seeking and learning and must complete 8 h of learning tasks monthly. The department head randomly selects test questions from the system every month for testing, and the test results are included in the employee’s performance evaluation.

The enterprise has updated and maintained the frequently malfunctioning equipment and systems identified through cluster analysis of group-level, unit-level, and workshop-level accidents, such as induced draft fans, compressors, and power systems. In particular, the frequency of inspection and maintenance has been increased for critical equipment and components, and a comprehensive daily, weekly, monthly, and quarterly inspection and maintenance system has been developed. Finally, after addressing all local risks, the enterprise has developed standardized operation manuals and institutional norms for personnel, equipment, materials, and environmental factors involved in the production process, forming a standard model based on scientific and rigorous work processes and long-term accumulated experience and habits, forming a deeply rooted code of conduct in the minds of members, minimizing uncontrollable factors, and effectively helping the enterprise maintain a high level of safety.

#### Implementation effect

**Fig. 11:**
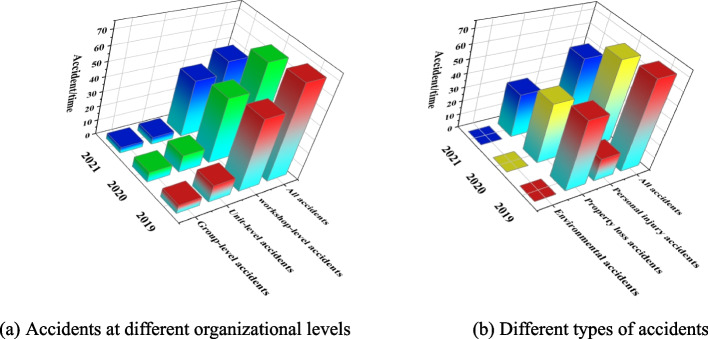
Comparative analysis of the number of accidents

The safety situation of the enterprise has been dramatically improved after rectification measures. We have statistics on the accidents in 2021, as shown in Fig. 11. The accidents in 2021 have decreased by 29% and 25% compared with 2019 and 2020. Compared with 2020, workshop-level, unit-level, and group-level accidents decreased by 13%, 58%, and 43%, respectively. From the perspective of accident types, personal injury and property loss accidents decreased by 19% and 27%, respectively, compared with 2020, and no environmental accident occurred in two years. The accidents have been effectively reduced by formulating measures to solve the two main risk factors of unsafe behaviors, such as weak safety awareness of employees, illegal operation, and frequent equipment damage.

## Discussion

The text cluster analyses of workshop-level, unit-level, and group-level accidents are conducted. The result indicates that the accidents of the sample enterprises are mainly personal injury accidents, production accidents, environmental pollution accidents, and quality accidents. The leading causes of personal injury accidents are employees’ unsafe behaviors, such as poor safety awareness, non-standard operation, illegal operation, untimely communication, etc. The leading causes of production accidents, environmental pollution accidents, and quality accidents include the unsafe state of materials, such as equipment damage, pipeline leakage, short-circuiting, excessive fluctuation of process parameters, etc. However, some accidents occur under the combined actions of unsafe human behavior and an unsafe material state. For example, employees are suddenly injured by steam pipe network leakage during work, and the operation is not standardized during maintenance, which further leads to excessive fluctuation of process parameters and equipment shut-down.

The risk factors of the enterprise can be effectively identified through accident cluster analysis. Enterprises should strengthen the training of employees’ safety knowledge and professional skills, improve the assessment mechanism, and enhance employees’ safety awareness and familiarity with the operation process to reduce employees’ unsafe behaviors. Given the unsafe state of objects, a more reasonable inspection and maintenance process system should be formulated, and defective equipment and accessories should be replaced in time. In particular, it is necessary to study the maintenance of the frequently faulty equipment identified above, such as the induced draft fan, motor, compressor, distribution room, and substation.

Although workshop-level accidents occur most frequently, the adverse effects are minor, and the hidden risks can be easily eliminated by adopting a series of improvement measures. Due to the small number of group-level accidents, the poor effect of cluster analysis, and the unclear division of accident types and causes, the accident triangle at the organization level can make up for this defect. So, as the number of unit-level accidents is positively related to group-level accidents, the hidden dangers of unit-level accidents can be solved, thus reducing and avoiding group-level accidents.

## Conclusion

This article improved the accident triangle and divided chemical accidents into group-level, unit-level, and workshop-level. Based on 484 accident reports of a large chemical enterprise in China, the Spearman correlation coefficient method was used to analyze the rationality of the accident classification. Based on accident classification, TF-IDF and K-means algorithms are used to extract keywords and text clustering analysis is carried out for accidents at all levels. The main conclusions are as follows:

1. Compared with the traditional accident classification method, the accident triangle proposed in this paper based on the organizational level dramatically reduces the differences between accidents, helps enterprises quickly identify risk factors, and prevents accidents.
2. There is a significant positive correlation between the unit-level and the group-level accident rate. Enterprises can prevent group-level accidents by reducing the number of unit-level accidents, which solves the problem that it is difficult to find the causes of group-level accidents due to the small number.
3. This method has achieved significant results in the one-year application process in a large chemical enterprise. Compared with 2020, workshop level, unit level, and group level accounts have been increased by 13%, 58%, and 43%, respectively. In the future, more enterprise data should be selected to validate this method.

### Supplementary Information

**Additional file 1.** Classification criteria for accidents at different levels.

**Additional file 2.** Example of accident report.

**Additional file 3.** Workshop-level accident clustering results.

## Data Availability

The datasets used and/or analysed during the current study available from the corresponding author on reasonable request.

## Abbreviations

OGP: International Association of Oil and Gas Producers
KDT: Knowledge Discovering in Texts
NLP: Natural Language Processing
TF-IDF: Term Frequency Inverse Document Frequency
