# Supplementary material for: A chemical accident cause text mining method based on improved accident triangle
Source: BMC Public Health. 2024 Jan 2;24:39. doi: 10.1186/s12889-023-17510-w (PMC10762847; doi:10.1186/s12889-023-17510-w)
Supplement: Supplementary file 1 — Additional file 1. Classification criteria for accidents at different levels. [file 12889_2023_17510_MOESM1_ESM.docx]

**Additonal file 1** Classification criteria for accidents at different levels

1. Workshop-level accidents refer to safety accidents within the production equipment or workshop scope and are mainly handled by workshop or on-site operators for emergency response. If necessary, the workshop leader can request assistance from various emergency teams of the company. If one of the following conditions is met, it is a workshop-level accident.

(1) One person is slightly injured in an accident.

(2) The direct economic loss caused by accidents is less than 100,000 yuan.

(3) The material loss caused by accident is less than 1 ton.

(4) One set of items of equipment is shut down due to one accident, and the daily output is decreased by no more than 50%, or two sets of equipment are shut down due to one accident, and the daily output is decreased by 25% - 50%.

(5) Unscheduled production affects normal production for less than 8 hours.

2. Unit-level accidents refer to safety accidents that cause significant harm and threat to company production and personnel, may cause casualties, property damage, and environmental damage, and cannot be controlled by the workshop’s rescue forces. They require the assistance of higher-level units or relevant rescue forces for disposal. If one of the following conditions is met, it is a unit-level accident.

(1) One accident causes two or more minor injuries, no severe damage, and death.

(2) The direct economic loss caused by accidents is more than 100,000 yuan and less than 500,000 yuan.

(3) The material loss caused by accident is more than 1 ton and less than 5 tons.

(4) One set of equipment is shut down due to one accident, and the output of 1-2 days is reduced by 100%, or two sets of equipment are shut down due to one accident.

(5) Unscheduled production affects the normal production time from 8 hours to less than 16 hours.

3. Group-level accidents are safety accidents that cause significant harm and threaten the company’s production and personnel safety, seriously affecting the surrounding environment and personnel safety and requiring external emergency rescue and response. If one of the following conditions is met, it is a group-level accident.

(1) One accident causes severe injury or death of 1 person or more.

(2) The direct economic loss caused by the accident is more than 500,000 yuan.

(3) The material loss caused by one accident is more than 5 tons.

(4) One set of equipment is shut down due to an accident, and the output for three days or more is decreased by 100%, or two sets of equipment are shut down due to one accident, and the output for two days or more is decreased by 100%, or one accident causes the shutdown of three or more sets of equipment, and the daily output is decreased by 100%.

(5) Unscheduled production affects the normal production time of more than, or equal to, 16 hours.
