# Supplementary material for: A chemical accident cause text mining method based on improved accident triangle
Source: BMC Public Health. 2024 Jan 2;24:39. doi: 10.1186/s12889-023-17510-w (PMC10762847; doi:10.1186/s12889-023-17510-w)
Supplement: Supplementary file 2 — Additional file 2. Example of accident report. [file 12889_2023_17510_MOESM2_ESM.docx]

**Additional file 2** Example of accident report

| No. | Accident position | Accident  time | Accident  level | Accident  nature | Consequence degree | Accident Process |
| --- | --- | --- | --- | --- | --- | --- |
| 1 | The second platform at the side of the 7# coke oven is at the 34# furnace pillar | 2020/3/7 | Unit Level | Injury | Minor injuries | On March 7, 2020, Shift 4 of the coking workshop of the coking plant used the coke oven maintenance time from 20:00 to 21:00 in the middle shift to paint and brighten the 18-35# furnace column in the contract area on the machine side of the team 7# coke oven. The operators used a bamboo rod with about 4.5 m as a painting tool. At about 21:21, when painting the lower part of the 34# furnace column, Yang accidentally fell from the second platform on the machine side because he faced the furnace column and faced the edge with his back. |
| 2 | Coke oven gas pipeline valve 2-10 | 2020/7/11 | Group level | Injury | Death | At 8:17 on July 11, 2020, the operator received a notice from the fine chemical plant that a blind plate needed to be added behind the 2-10 gas pipeline valve due to the overhaul of the giant flare. At 9:40, after the bills were handled, four people from the fine chemical plant were arranged to carry an explosion-proof 50 percussion wrench to the pipe rack for blind plate-adding operation. At 13:00, the operator put the blind plate into the flange and found that the blind plate pad was deformed. The operator Hu contacted the crane to prepare to adjust the blind scale. At 13:19, a flash explosion occurred when the vertical ladder approached the ground, which struck the operator Yang on the operation platform, pushing him from the platform to the ground. The on-site personnel called the police. The fire truck cooled the coke oven gas pipeline, and the ambulance sent the three injured to the hospital for rescue. Yang died due to ineffective rescue, and the other two operators were in recovery with initial symptoms and extensive burns. |
| … | … | … | … | … | … | … |
