# Supplementary material for: A chemical accident cause text mining method based on improved accident triangle
Source: BMC Public Health. 2024 Jan 2;24:39. doi: 10.1186/s12889-023-17510-w (PMC10762847; doi:10.1186/s12889-023-17510-w)
Supplement: Supplementary file 3 — Additional file 3. Workshop-level accident clustering results. [file 12889_2023_17510_MOESM3_ESM.docx]

**Additional file 3** Workshop-level accident clustering results

| No. | Accident record content | Keywords | Cluster no. |
| --- | --- | --- | --- |
| 1 | On February 10, Wang, an employee of the coking plant, crushed his right thumb between the coal groove of the 3# ballast tamper and the guide groove machine in coking workshop 1. | Coking plant, coking, workshop, ballast tamper, coal groove, crush injury, right thumb | 0 |
| 2 | At 9:10 a.m. on September 2, Ji, the operator of the anchor mesh section, was processing the bolt thread on the thread rolling machine. Because the bolt head was not rolled straight, the force was uneven during thread rolling and suddenly bounced up, hitting his right ring finger, and causing cracking of the middle joint of the ring finger. | Anchor net, workshop section, operator, thread rolling machine, processing, bolt, roll straight, bounce, hit, right hand, ring finger, joint, crack | 0 |
| 3 | Due to the tripping of the cooling fan of the frequency conversion cabinet, the wind pressure difference in the cabinet is less than the set value, triggering the early warning, and causing the tripping of the gear pump and the interlocking tripping of the extrusion granulator. | Fan, trip, cabinet, wind pressure, differential pressure, less than, set value, trigger, early warning, gear pump, extrusion granulator, interlocking, shutdown | 1 |
| 4 | At 16:46 on November 13, 2016, 3# furnace operating conditions were abnormal, and the deviation between feed water and evaporation was significant. Through inspection, the economizer was found to be leaking. | Operating conditions, abnormal, feed water, evaporation, deviation, large, inspect, economizer, leak | 2 |
| 5 | At 4:16 on October 1, 2015, the circulating hydrogen compressor k-1101b in the compressor room suddenly tripped, resulting in the interlocking tripping of the heating furnace and the high-speed pump. After an on-site investigation, the safety barrier output temperature fluctuated wildly due to the oxidation of the safety barrier wiring and the failure of the indicator light, causing the tripping of the circulating hydrogen compressor owing to the high stator temperature of the electric machinery. | Compressor, trip, heating furnace, high-speed pump, interlocking, check, safety barrier, connection, oxidation, indicator, light, output, temperature, fluctuate, large, electric machinery, stator. | 3 |
| 6 | At 19:00 on January 22, 2016, the coal quantity in the 2# pulverized coal pipeline of the 3# gasifier began to fluctuate or even cut off. After repeating back blowing, it was found to be ineffective. Through inspection, there were sundries in the angle valve. After cleaning, the coal quantity in the pulverized coal pipeline was regular. | Gasifier, pulverized coal, pipeline, coal quantity, fluctuate, check, clean, angle valve, sundries. | 4 |
| … | …… | …… | …… |
